# Global land use for 2015–2100 at 0.05° resolution under diverse socioeconomic and climate scenarios

**DOI:** 10.1038/s41597-020-00669-x

**Published:** 2020-10-02

**Authors:** Min Chen, Chris R. Vernon, Neal T. Graham, Mohamad Hejazi, Maoyi Huang, Yanyan Cheng, Katherine Calvin

**Affiliations:** 1 Joint Global Change Research Institute, Pacific Northwest National Laboratory, 5825 University Research Ct., Suite 3500, College Park, MD 20740 USA; 2 Atmospheric Sciences and Global Change Division, Pacific Northwest National Laboratory, P.O. Box 999, Richland, WA 99352 USA; 3 Present Address: Office of Science and Technology Integration, National Weather Service, National Oceanic and Atmospheric Administration, Silver Spring, MD USA

**Keywords:** Climate and Earth system modelling, Projection and prediction

## Abstract

Global future land use (LU) is an important input for Earth system models for projecting Earth system dynamics and is critical for many modeling studies on future global change. Here we generated a new global gridded LU dataset using the Global Change Analysis Model (GCAM) and a land use spatial downscaling model, named Demeter, under the five Shared Socioeconomic Pathways (SSPs) and four Representative Concentration Pathways (RCPs) scenarios. Compared to existing similar datasets, the presented dataset has a higher spatial resolution (0.05° × 0.05°) and spreads under a more comprehensive set of SSP-RCP scenarios (in total 15 scenarios), and considers uncertainties from the forcing climates. We compared our dataset with the Land Use Harmonization version 2 (LUH2) dataset and found our results are in general spatially consistent with LUH2. The presented dataset will be useful for global Earth system modeling studies, especially for the analysis of the impacts of land use and land cover change and socioeconomics, as well as the characterizing the uncertainties associated with these impacts.

## Abstract (summary)

**Table:** 

Measurement(s)	land use process • global land use and land cover
Technology Type(s)	computational modeling technique
Factor Type(s)	year of data collection • Shared Socioeconomic Pathways scenarios • Representative Concentration Pathways scenarios
Sample Characteristic - Environment	land
Sample Characteristic - Location	Earth (planet)

Machine-accessible metadata file describing the reported data: 10.6084/m9.figshare.12967760

## Background & Summary

Land use (LU) change represents one of the most important human effects on the Earth system^1,2^, with profound physical and biogeochemical impacts at both regional and global scales. Therefore, existing Earth system models (ESMs) widely take land use records as a key input for more realistically simulating Earth system dynamics and analyzing the effects of LU on climate and biogeochemical cycling. For example, the Land Use Model Intercomparison Project (LUMIP) has been launched to advance understanding of the impacts of LU on climate^3^ in the Climate Model Intercomparison Project Phase 6 (CMIP6)^4^.

ESMs have been found to be sensitive to LU changes^5–7^. Therefore, uncertainties in LU datasets can lead to a propagation of uncertainty in ESM projections that have to be carefully considered and evaluated. This could be relatively more straightforward for historical land use and land cover change, which can be benchmarked by observational datasets such as those based on by ground investigation or satellite remote sensing^8,9^. In contrast, it is challenging to evaluate the uncertainties of future LU projections, since there is no benchmarking reference. One possible approach for this purpose is to compare multiple LU projections from different models, following the similar philosophy of the model intercomparison projects^3,6^.

To date, there are still limited global gridded LU datasets that are publicly available for ESM simulations. One representative example is the Land Use Harmonization dataset version 2 (LUH2)^10^, which was produced to provide gridded LU data annually at 0.25° × 0.25° resolution for the historical reconstructions and under future scenarios (850–2300). Essentially, LUH2 harmonizes and at times downscales LU projections from the Global Change Assessment Model (GCAM) or similar models, such as IMAGE^11^, REMIND-MAGPIE^12^, MESSAGE-GLOBIOM^13^, and AIM^14^. LUH2 data has been produced for a small set of scenarios, e.g., a subset of combinations of Shared Socioeconomic Pathways (SSPs)^15^ and Representative Concentration Pathways (RCPs)^16^ chosen by the Scenario Model Intercomparision Project (ScenarioMIP)^17^ for CMIP6 experiments, although the full set of SSP-RCP combinations can be used to examine how different levels of radiative forcing targets can be achieved in 2100 under different underlying socioeconomic pathways, and represent the most updated understanding and design of future pathways of socioeconomic development and greenhouse gas emissions^18^. In addition, LUH2 classifies the land into five land use states (cropland, pasture, primary, secondary and urban) and 12 sub-states, in a classification system by emphasizing human activities (e.g., primary vs. secondary). This classification system cannot be directly used in many of the land models of ESMs, which typically use plant functional types (PFTs)^19–21^, with detailed classification according to the physical, phylogenetic and phenological characteristics of the land covers. For example, one of the most widely used land surface models, the Community Land Model 5 (CLM5) in the Community Earth System Model 2 (CESM 2) uses 79 PFTs^22^ to capture the diversity of land surface processes for different land covers. Thus, a LU dataset with similar level of diversity of land cover types and following the concept of PFT is useful for ESM simulations and the followed modeling analysis.

**Fig. 1:**
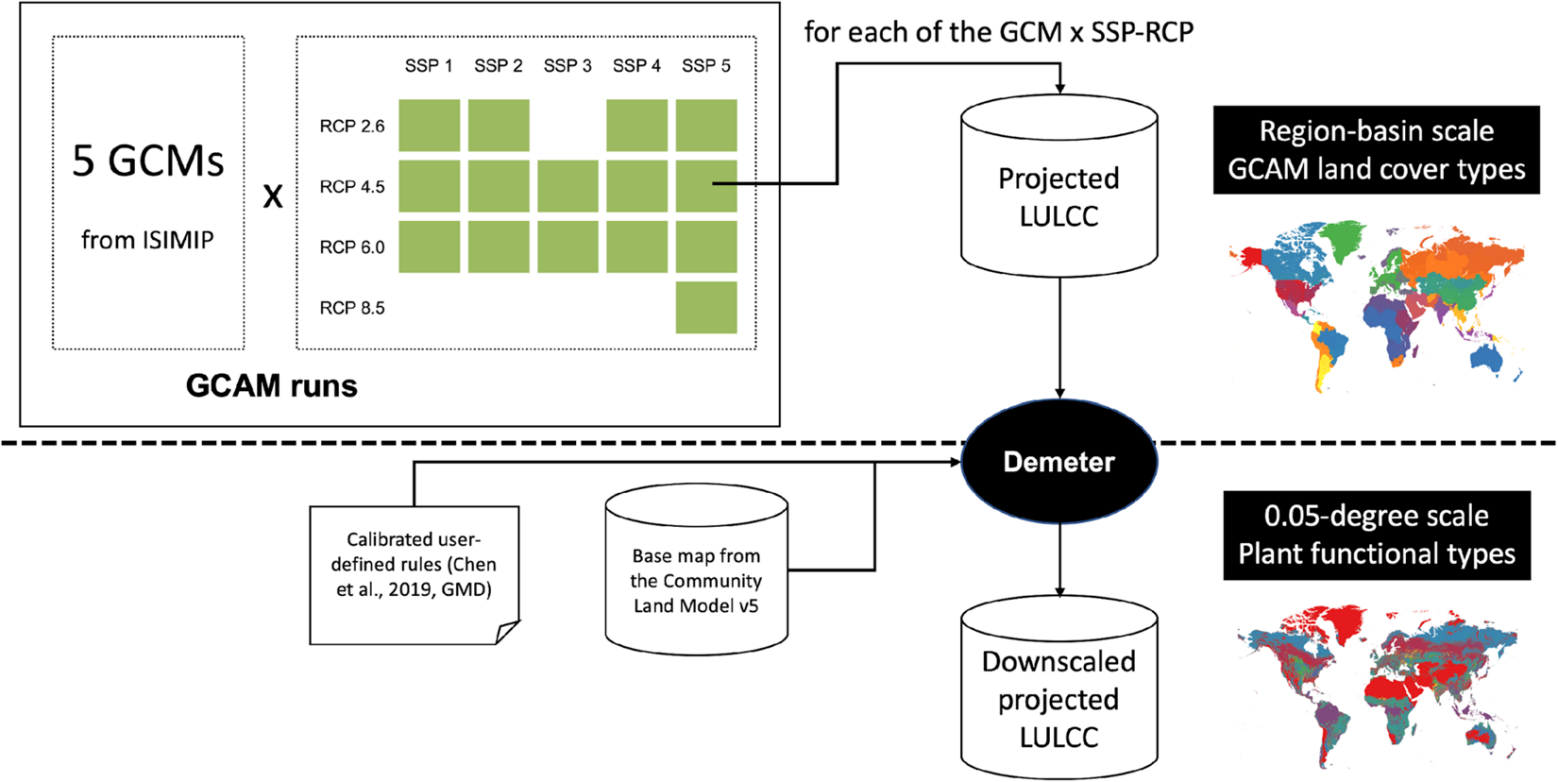
A schematic flow chart of GCAM projections of global land use and land cover change in the 21st century and downscaling of these projections into gridded products using Demeter.

GCAM has been used to explore future societal and environmental scenarios under different climate scenarios^23,24^, including land use^25–27^. Here we started from GCAM v4.3, but incorporated water basin level modeling of water supply and demands^28^, distinctions between renewable and nonrenewable water sources^29^, and socioeconomic scenario specific water demand responses^30^, and used it to provide LU projections at the intersection of geopolitical regions and water basins at 5-year time step^31^ (Fig. 1). Those updates were later incorporated into the current GCAM v5 releases. Furthermore, the recent development of a spatial disaggregation model (Demeter)^32,33^ enables downscaling GCAM’s regional LU to variable grid scales. Similar to LUH2 algorithms, Demeter can be applied for downscaling any regional LU projections from any model in any period but is more flexible in terms of the output spatial resolution and land cover classification. Demeter has been carefully parameterized and applied to continental and global LU downscaling. The dataset presented here includes a Demeter-downscaled projection of global LU from GCAM at 0.05° × 0.05° resolution over 2015–2100 under 15 plausible SSP-RCP scenarios^31^. Rather than using broad land cover types^10,32,34^, the dataset classifies land cover into 32 PFTs that are typically used in existing ESMs.

The dataset provides an alternative and more comprehensive future gridded LU product for ESMs. We anticipate that the dataset will be widely used by researchers in ecological modeling, Earth system modeling, land-atmosphere interactions, agriculture, energy market, water resources management, and socioeconomic analysis, to investigate the complex feedbacks between each of these Earth system components, and better understand the role of human activities in these processes.

## Methods

### GCAM projections of LU under SSP-RCPs

GCAM links land use and land cover change, socioeconomics, the water sector, energy system, and climate in a market-equilibrium system that allows for prices to be adjusted within each time step (generally 5 years) to ensure that the supply and demand of goods and services remains balanced. In our GCAM runs, the model divides the global land area into 32 geopolitical regions, or 229 water basins. The intersection between the geopolitical regions and water basins results in 384 basic GCAM spatial units (region-basin). GCAM projects the evolution of the LU mix with up to 39 land cover types (hereafter refer to GCAM Land cover Types, or GLTs, see Table 1). Note that the GLTs are primarily classified according to ag-economic sectors and from the land use perspective, thus have to be reconciled to user-defined final land types (FLTs) which are directly applicable to ESMs with user-defined rules^34^.

**Table 1:** The GCAM Land Types (GLTs).

GLT #	GCAM Land Types	GLT #	GCAM Land Types
1	Corn: irrigated	21	Biomass grass: irrigated
2	Corn: rainfed	22	Biomass grass: rainfed
3	Wheat: irrigated	23	Biomass tree: irrigated
4	Wheat: rainfed	24	Biomass tree: rainfed
5	Rice: irrigated	25	Misc-crop: irrigated
6	Rice: rainfed	26	Misc-crop: rainfed
7	Root tuber: irrigated	27	Other arable land
8	Root tuber: rainfed	28	Palm fruit: irrigated
9	Oil crop: irrigated	29	Palm fruit: rainfed
10	Oil crop: rainfed	30	Pasture
11	Sugar crop: irrigated	31	Unmanaged pasture
12	Sugar crop: rainfed	32	Urban land
13	Other grain: irrigated	33	Willow
14	Other grain: rainfed	34	Forest
15	Fiber crop: irrigated	35	Unmanaged forest
16	Fiber crop: rainfed	36	Shrubland
17	Fodder grass: irrigated	37	Grassland
18	Fodder grass: rainfed	38	Tundra
19	Fodder herb: irrigated	39	Rock, Ice, Desert
20	Fodder herb: rainfed		

Consistent with the design of CMIP6, this dataset was developed under scenarios with both varying socioeconomics and climate systems, represented by SSPs and RCPs, respectively^17^. The RCPs are a set of four future greenhouse gas emission pathways in which end-of-century radiative forcing approaches four-levels (2.6, 4.5, 6.0, and 8.5 W m^2^) by altering future greenhouse gas emissions and by changing underlying socioeconomic projections^16^. The SSPs are a set of five future scenarios (SSP1 to SSP5) which were designed to span a range of future socioeconomic conditions, and can be used in combination with the RCPs. SSPs look at different ways in which the world might evolve in the future. Specifically, SSP1 represents a world focusing on sustainable growth and equality with low challenges to mitigation and adaptation (“taking the green road”); SSP2 represents a world where trends broadly follow their historical patterns with medium challenges to mitigation and adaptation (“a middle of the road”); SSP3 is a fragmented world of resurgent nationalism with high challenges to mitigation and adaptation (“regional rivalry – a rocky road”); SSP4 represents a world of increasing inequality with low challenges to mitigation but high challenges to adaptation (“inequality – a road divided”); and SSP5 represents a world of rapid and unconstrained growth in economics and energy use with high challenges to mitigation and low challenges to adaptation (“fossil-fueled development”). Broadly speaking, land use is strongly regulated under SSP1, mediumly regulated under SSP2, limitedly regulated under SSP3, strongly regulated in middle/high income countries but limitedly regulated in low income countries under SSP4, and medium regulated under SSP5^35^.

The SSPs and RCPs combinations form a set of future global change scenarios which provide the basis for the next round of CMIP6 and future Intergovernmental Panel on Climate Change (IPCC) assessments^4,17^. In total, these combinations create a set of fifteen potential future scenarios^31^ which are varied five times, for using bias-corrected precipitation and temperature data from five global climate models (GCMs, including GFDL-ESM2M, HadGEM2-ES, IPSL-CM5A-LR, MIROC5 and NorESM-M) from the Inter-Sectoral Impact Model Intercomparison Project (ISIMIP)^36^ to drive the estimation of water supply, hydropower capacity changes, and building energy demands in GCAM under each RCP scenario. Details are provided in Graham *et al*.^31^. Uncertainties due to the use of the five GCMs are provided with the dataset. Therefore, these GCAM runs have resulted in 75 projections of global (excluding the Antarctic) LU at region-basin level for every five years over 2015 to 2100 (Fig. 1).

### Downscale GCAM-projected land use with demeter

We used Demeter, a spatial disaggregation model, to convert LU projections by GCAM from regional scale to grid cells. Detailed algorithms and optimization procedures are documented in several publications^32–34,37^. In brief, Demeter uses a base land cover map at a target resolution as the reference, and strategically allocates the projected area changes for each land type by models (e.g., GCAM) to the target grid cells, following a series of user-defined rules and spatial constraints.

Demeter requires converting the land cover classes defined in the parent model (i.e., GLTs) and the base map (classified in base land types (BLTs)) to user-defined final land types (FLTs). Downscaled land cover types are thus presented in FLTs. For the purpose of supporting Earth system modeling, we defined 32-type FLTs (Table 2) which were designed to balance and keep the most detailed land cover classification between the 39 GLTs (from GCAM) and 79 BLTs (from the Community Land Model version 5 (CLM5) (Table 3), the land component of the Community Earth System Model version 2 as one of the most widely-used ESMs), thus with sufficient plant functional characterization for use by ESMs. In particular, we adopted the base map from the 0.05° × 0.05° 79-BLT land cover data that has been used in CLM5 and represents the land cover in the early 21st century (e.g., 2005). The rules for converting GLTs and BLTs to FLTs are developed with the definitions of GLTs, BLTs and FLTs. and are provided in the input datasets at^28,29,32,35^.

**Table 2:** The final land types in the downscaled product.

FLT #	Final Land Types	Acronym	FLT #	Final Land Types	Acronym
1	Needleleaf evergreen tree – temperate	NET_tem	17	Wheat: rainfed	Wheat_rf
2	Needleleaf evergreen tree - boreal	NET_bor	18	Wheat: irrigated	Wheat_irr
3	Needleleaf deciduous tree – boreal	NDT_bor	19	Soybean: rainfed	Soy_rf
4	Broadleaf evergreen tree – tropical	BET_tro	20	Soybean: irrigated	Soy_irr
5	Broadleaf evergreen tree – temperate	BET_tem	21	Cotton: rainfed	Cotton_rf
6	Broadleaf deciduous tree – tropical	BDT_tro	22	Cotton: irrigated	Cotton_irr
7	Broadleaf deciduous tree – temperate	BDT_tem	23	Rice: rainfed	Rice_rf
8	Broadleaf deciduous tree – boreal	BDT_bor	24	Rice: irrigated	Rice_irr
9	Broadleaf evergreen shrub - temperate	BES_tem	25	Sugar crop: rainfed	Sugarcrop_rf
10	Broadleaf deciduous shrub – temperate	BDS_tem	26	Sugar crop: irrigated	Sugarcrop_irr
11	Broadleaf deciduous shrub – boreal	BDS_bor	27	Other crop: rainfed	OtherCrop_rf
12	C3 Arctic	C3_gra_arc	28	Other crop: irrigated	OtherCrop_irr
13	C3 Grass	C3_gra	29	Bioenergy crop: rainfed	Bioenergy_rf
14	C4 Grass	C4_gra	30	Bioenergy crop: irrigated	Bioenergy_irr
15	Corn: rainfed	Corn_rf	31	Urban	Urban
16	Corn: irrigated	Corn_irr	32	Barren	Barren

**Table 3:** Base Land Types (BLTs) as used in Community Land Model version 5 as the base map for downscaling.

BLT #	Base Land Types	BLT #	Base Land Types
1	Non-vegetated	41	irrigated coffee
2	Needleleaf evergreen tree – temperate	42	rainfed cotton
3	Needleleaf evergreen tree - boreal	43	irrigated cotton
4	Needleleaf deciduous tree – boreal	44	rainfed datepalm
5	Broadleaf evergreen tree – tropical	45	irrigated datepalm
6	Broadleaf evergreen tree – temperate	46	rainfed foddergrass
7	Broadleaf deciduous tree – tropical	47	irrigated foddergrass
8	Broadleaf deciduous tree – temperate	48	rainfed grapes
9	Broadleaf deciduous tree – boreal	49	irrigated grapes
10	Broadleaf evergreen shrub - temperate	50	rainfed groundnuts
11	Broadleaf deciduous shrub – temperate	51	irrigated groundnuts
12	Broadleaf deciduous shrub – boreal	52	rainfed millet
13	C3 Arctic	53	irrigated millet
14	C3 Grass	54	rainfed oilpalm
15	C4 Grass	55	irrigated oilpalm
16	c3 unmanaged rainfed crop	56	rainfed potatoes
17	c3 unmanaged irrigated crop	57	irrigated potatoes
18	rainfed temperate corn	58	rainfed pulses
19	irrigated temperate corn	59	irrigated pulses
20	rainfed spring wheat	60	rainfed rapeseed
21	irrigated spring wheat	61	irrigated rapeseed
22	rainfed winter wheat	62	rainfed rice
23	irrigated winter wheat	63	irrigated rice
24	rainfed temperate soybean	64	rainfed sorghum
25	irrigated temperate soybean	65	irrigated sorghum
26	rainfed barley	66	rainfed sugarbeet
27	irrigated barley	67	irrigated sugarbeet
28	rainfed winter barley	68	rainfed sugarcane
29	irrigated winter barley	69	irrigated sugarcane
30	rainfed rye	70	rainfed sunflower
31	irrigated rye	71	irrigated sunflower
32	rainfed winter rye	72	rainfed miscanthus
33	irrigated winter rye	73	irrigated miscanthus
34	rainfed cassava	74	rainfed switchgrass
35	irrigated cassava	75	irrigated switchgrass
36	rainfed citrus	76	rainfed tropical corn
37	irrigated citrus	77	irrigated tropical corn
38	rainfed cocoa	78	rainfed tropical soybean
39	irrigated cocoa	79	irrigated tropical soybean
40	rainfed coffee		

In addition, Demeter also requires a set of user-defined rules and spatial constraints for the downscaling processes, including (1) treatment order, which decides the order of allocating changes among land cover types; (2) transition priority, which defines the order of transitions among land cover types; and (3) spatial constraints, such as the spatial distributions of soil workability and nutrient availability. Here we followed the treatment order and transition priority for broader land cover types (e.g., forest, shrub, grass, crop, urban. snow and sparse) as suggested in Chen *et al*.^32^, but with additional considerations on the variation inside the broad land cover types for the transition priorities. Transition priority is determined by geographical co-occurrence across FLTs. The co-occurrence is defined in four dimensions (from the highest to lowest importance): climate zones (temperate, boreal and tropical), leaf shapes (needleleaf and broadleaf), phenology types (evergreen and deciduous), and vegetation types (trees, grassland, shrubland, cropland, etc.). For example, a ‘temperate needleleaf evergreen forest’ is more likely transitioned to a ‘temperate broadleaf evergreen shrub’ than a ‘boreal needleleaf evergreen forest’, and or a ‘temperate broadleaf deciduous forest’. We used the same spatial constraints as published in Le Page *et al*.^34^, and Chen *et al*.^32^, but have linearly interpolated the spatial weighting for soil workability and nutrient availability data into 0.05° to be consistent with our target downscaling resolution. In addition, a couple of key parameters in Demeter have been identified with the most importance for governing the downscaling processes^18^, including: (1) *r*, the ratio of allocating land cover change as intensification (i.e., increasing a particular land cover in a grid cell in which it already exists); and (2) *τ*, a threshold percentage of suitable grid cells (determined by spatial constraints) to accept extensified land cover change allocation (i.e., creating new land cover in grid cells in which it does not yet exist). Within this study, we adopted the global optimal value of *r* = 1 and *τ* = 0.6 based on our earlier calibration^18^. Detailed rules and constraints are included along with the other input data, such as GCAM projections under the fifteen SSP-RCP scenarios driven by the five GCMs and the base map for downscaling, are archived at as a publicly available dataset^38^ (Table 4).

**Table 4:** Summary of the Inputs for producing the presented dataset.

Type	Description	Spatial scale	Source
Input data	GCAM projections of land use	Region-basin	GCAM runs under 15 SSP-RCP scenarios^31^
	Base map	0.05°	Community Land Model^22^
Land type harmonization	GCAM Land Types (GLTs) to Final Land Types (FLTs)	Not available	Definitions of GLTs, BLTs and FLTs
	Base Land Types (BLTs) to Final Land Types (FLTs)		
Disaggregation rules/parameters	Treatment order	Not available	Previous studies^32,34^
	Transition priority		
	Key parameters		
	Spatial constraints	0.05° (resampled from 30 arc-second)	the Harmonized world soil database v1.2 (publicly available at http://www.fao.org/soils-portal/soil-survey/soil-maps-and-databases/harmonized-world-soil-database-v12/en/, accessed April 10, 2020)

These inputs are publicly available ^38^.

However, it is possible that the definitions of a land type may be different in GCAM and the base map, even though their names are similar^39,40^. For example, grassland and shrubland of GCAM are more broadly defined than in the base map, thus some barren area (as defined in BLTs) are classified as grassland/shrubland in GCAM. Such difference may result in inconsistency between the downscaled results and the base map. Although the existing conversion rules may work within each broad land type (i.e., forests, grassland and shrubland, cropland, bioenergy, urban, and barren), there is no reasonable way to decide the conversion between two distinct broad land types (e.g., grassland vs. barren) according to the existing GLT-to-FLT conversion rules in Demeter. For example, it is reasonable to convert a GLT of ‘Grassland’ to a FLT of ‘C3 grass’, but not reasonable to convert ‘Grassland’ to ‘Barren’ in Demeter’s conversion rule. To address these potential inconsistencies, we developed a preprocessing procedure to harmonize GCAM projections to be more consistent with the base map in two steps. Such a procedure can be used to match any base map, ensuring consistency between historic and future land use for any user of the product (e.g., an ESM). The essential idea of the procedure is to adjust the area of each GLT in GCAM projections at the starting point (governed by the CLM5 base map, i.e., in 2005) to match the base map (Step 1) but keep the fractional change of each GLT as projected by GCAM in the following time steps (Step 2).

*Step 1. Harmonize GCAM projection in year 2005*. We used Eq. (1) to adjust the GCAM-projected area for each GLT in each GCAM’s spatial unit (region-basin) to match that according to the base map:

1 $${A}_{GLT,u,H}={A}_{u,G}\times \frac{{A}_{BT,u,B}}{{A}_{u,B}}\times \frac{{A}_{GLT,u,G}}{{A}_{BT,u,G}}$$

where *A*_*GLT,u,H*_ is the harmonized (*H*) GLT area in region-basin *u*. *A*_*u,G*_ is the area of region-basin *u* in the original GCAM projection, *A*_*BT,u,B*_ is the area of a broad land type *BT* (i.e., forests, grassland and shrubland, cropland, bioenergy, urban, and barren) in *u* according to the base map (*B*), and *A*_*u,B*_ is the area of region-basin *u* according to the base map. Thus, *A*_*BT,u,B*_*/A*_*u,B*_ gives the areal fraction of broad land type *BT* in region-basin *u* according to the base map. *A*_*GLT,u,G*_ is the GCAM-projected area of a GLT in region-basin *u*, and *A*_*BT,u,G*_ is the GCAM-projected area of a broad land type *BT* in region-basin *u*. Thus *A*_*GLT,u,G*_ /*A*_*BT,u,G*_ gives the areal fraction of a GLT to its associated broad land type *BT* in region-basin *u* according to GCAM projection. If *A*_*BT,u,G*_ = 0, i.e., a *BT* does not exist in *u* according to GCAM projection (e.g., bioenergy plants), *A*_*GLT,u,H*_ is set to be 0.

*Step 2. Harmonize GCAM projections for each time step after 2005*. Harmonized area for each GLT in region-basin *u* at time step *t* (*t* = 2010, 2015, …, 2100) is calculated as *A*_*GLT,u,H,t*_ = *A*_*GLT,u,H,t-1*_ *· A*_*GLT,u,G,t*_ /*A*_*GLT,u,G,t-1*_. If *A*_*GLT,u,G,t-1*_ = 0 (e.g., no bioenergy classification in 2005), we first set *A*_*GLT,u,H,t*_ to be *A*_*GLT,u,G,t*_. Then a scaling factor *f* is applied to adjust area of other land types in each region-basin *u*. *f* is calculated as the fraction of the area of GLTs for which *A*_*GLT,u,G,t-1*_ > *0* in *u*.

The preprocessing procedure produces a harmonized GCAM projections to be used in Demeter. In this data descriptor, we primarily present the downscaled LU datasets from the harmonized GCAM projections after applying the preprocessing procedure. But we provide both the downscaled results from the harmonized and original GCAM projections in our dataset. The former one better matches the base map with the PFT-oriented land type definition; and the latter is more consistent with the original GCAM projections. If there is no special declaration, ‘our dataset’ is referred to the results from the harmonized GCAM projection in this paper.

## Data Records

The dataset includes the projected global gridded land use (excluding the Antarctic) for the period of 2015–2100 at 0.05-degree resolution and 5-year time step under the fifteen SSP-RCP scenarios driven by five GCMs (Fig. 1). Note that we provide two versions of the dataset: one was developed from the harmonized GCAM projections, and the other one was developed from the original GCAM projections (see descriptions above). In each version of the dataset, the spatially explicit results driven by the five GCMs as well as their mean and standard deviation for each of the fifteen SSP-RCP scenarios are provided. All the data has been stored in self-describing NetCDF format which is commonly used by the ESM community. More specifically, the data in each year includes grid-explicit fraction (in percent) of each of the 32 final land types (Table 2) that are widely used in current ESMs. The gridded LU data can be directly downloaded at 10.25584/data.2020-07.1357/1644253^41^. The NetCDF files are named as “GCAM_Demeter_LU_V_SSP_RCP_Model_Year.nc”, where “V” could be “H” or “O”, denoting the two versions (using harmonized or original GCAM projections) of the dataset; “SSP” and “RCP” denote the SSP and RCP scenarios, “Year” denotes the year of the land use data, and “GCM” denotes the source driving forcing data from five GCMs, or the mean (“modelmean”) and the standard deviation (“modelstd”) of the results from the five GCMs.

## Technical Validation

### GCAM projected land use change

**Fig. 2:**
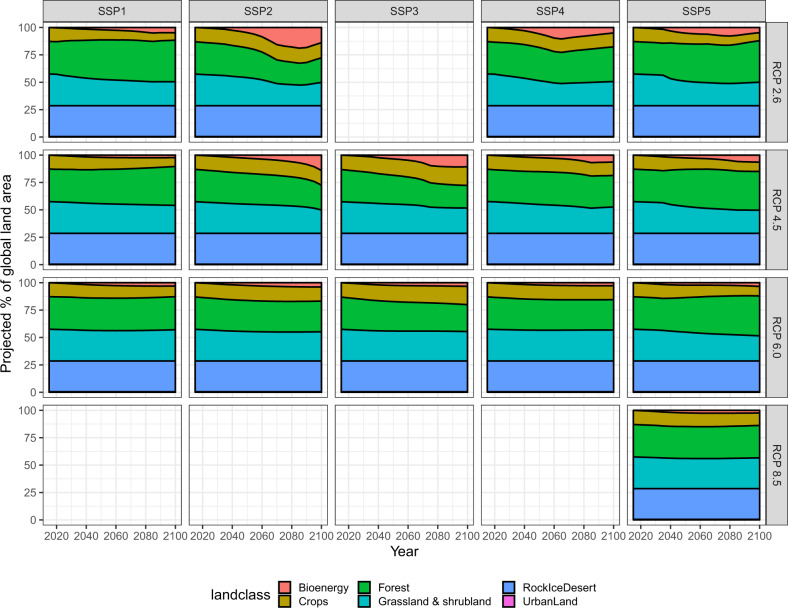
Global land use and land cover change projected by GCAM over 2015–2100 under the fifteen SSP-RCPs. We grouped the GCAM land types into a few broad land classes for illustration clarity as shown in the figure, including Bioenergy (GLT# 21–24), Crops (GLT# 1–20, 25–31, 33), Forest (GLT# 34, 35), Grassland & Shrubland (GLT# 36, 37, 38), RockIceDesert (GLT# 39), and Urbanland (GLT# 32). Note that Urbanland may be not visible because of its small area.

We examined the harmonized GCAM land use projections for producing this dataset (Fig. 2). Specifically, bioenergy land area will increase under all SSPs; crop area will decrease under SSP1, SSP4, and SSP5, but will increase under SSP2 and SSP3; forest area will increase under SSP1 and SSP5, but will decrease under SSP2 and SSP3; grassland and shrubland will generally decrease under all SSPs. The dynamic of urban land and barren (rock, ice, desert) are not projected by GCAM, thus they will remain unchanged over time. The projected land use areas have large variations across RCPs under each of the SSPs. For example, under all SSPs, the bioenergy plant area will increase (and accordingly the forest and grassland will decrease) much faster under RCP 2.6 than RCP 4.5 and 6.0, for the higher demand of clean energy to meet more strong limitation of greenhouse gas emission under RCP 2.6. These temporal trends of the GCAM projections generally match the qualitative descriptions of land use futures under SSPs in Popp *et al*.^35^.

### Spatial consistency with LUH2

**Fig. 3:**
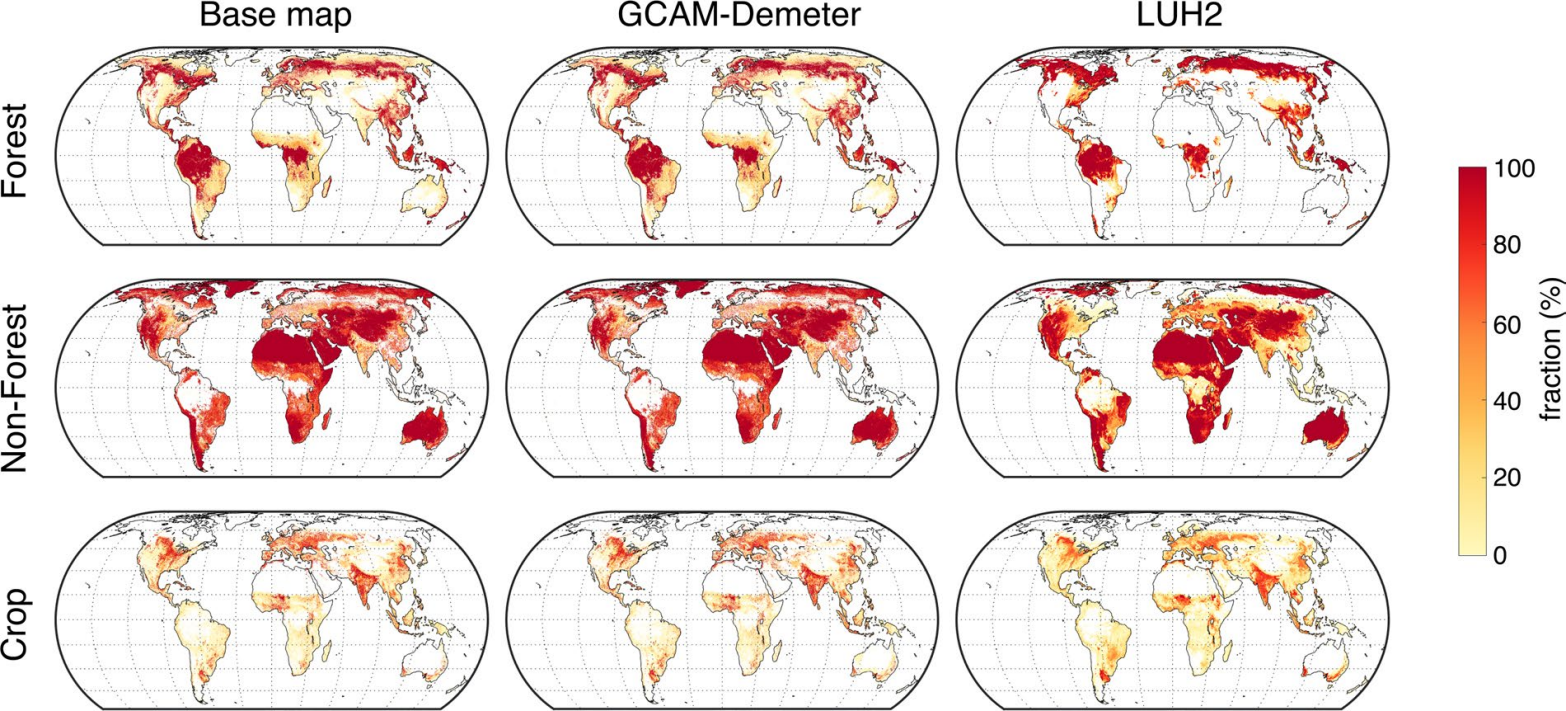
Comparison of land use spatial pattern between the base map, and our dataset and LUH2 in 2015. The land cover types have been grouped into broad types (Forest, Non-forest and Crop) to minimize the inconsistency between LUH2 types and the FLTs in our dataset. For LUH2, ‘Forest’ includes ‘primf’ and ‘secdf’, ‘Non-forest’ include ‘primn’, ‘secdn’, ‘pastr’ and ‘range’, and ‘Crop’ includes ‘c3ann’, ‘c4ann’, ‘c3per’, ‘c4per’ and ‘c3nfx’. Details can be found in the LUH2 dataset (https://luh.umd.edu/data.shtml). For our dataset, ‘Forest’ refers to the sum of FLT# 1–8, ‘Non-Forest’ refers to the sum of FLT# 9–14 and 32, and ‘Crop’ refers to the sum of FLT# 15–30; For the base map, ‘Forest’ refers to the sum of BLT#2–9, ‘Non-Forest’ refers to the sum of BLT# 1 and 10–15, and ‘Crop’ refers to the sum of BLT# 16–79.

We compared the land use patterns between the base map (representing the ‘truth’ in the early 21^st^ century), and our dataset and LUH2 data in 2015. Because the land cover classification is different between the our dataset and LUH2, we grouped them into three broad land cover types (‘Crop’, ‘Forest’, and ‘Non-forest’) for comparison, as shown in Fig. 3. Here we used the data from LUH2 and our dataset under the scenario SSP4 RCP6.0, because the source of land use information under SSP4-RCP6.0 in LUH2 only is from GCAM. In general, the two datasets show similar spatial variations of the three broad land cover types with minor inconsistencies within some regions. Our GCAM-Demeter-based dataset better matches the spatial patterns in the base map than the LUH2 data. The Pearson’s correlation coefficients between the base map and our dataset are 0.99 for all three broad types, while they are 0.70, 0.59, and 0.85 between the base map and LUH2 for ‘Forest’, ‘Non-forest’ and ‘Crop’, respectively. This is not surprising because the base map was used as the reference for downscaling, but it suggests that our downscaling approach is successful in producing accurate historical land cover as suggested by the base map, which serves a good starting point for generating gridded land use data in the future years. It should be noted that the comparison does not mean LUH2 is incorrect, because of LUH2 uses a different input data and strategy for spatial downscaling, and use different land cover definitions. Primary differences between our dataset and LUH2 are in the northern high latitudes. For example, while LUH2 does not classify Greenland as any of the three broad land cover types, our dataset shows Greenland as ‘Non-forest’, because GCAM classifies ‘Rock, Ice, Desert’ into a single type (Table 1). Both the base map and our dataset show some forest in Russia’s Kamchatka Peninsula, while LUH2 suggests dense forest coverage in that region, probably due to the definition of forest in LUH2, which is based on carbon density other than a specific PFT. Note that the spatial resolution is 0.25° and 0.05° for the LUH2 data and our dataset, respectively, thus our data shows more spatial variation than the LUH2 data.

**Fig. 4:**
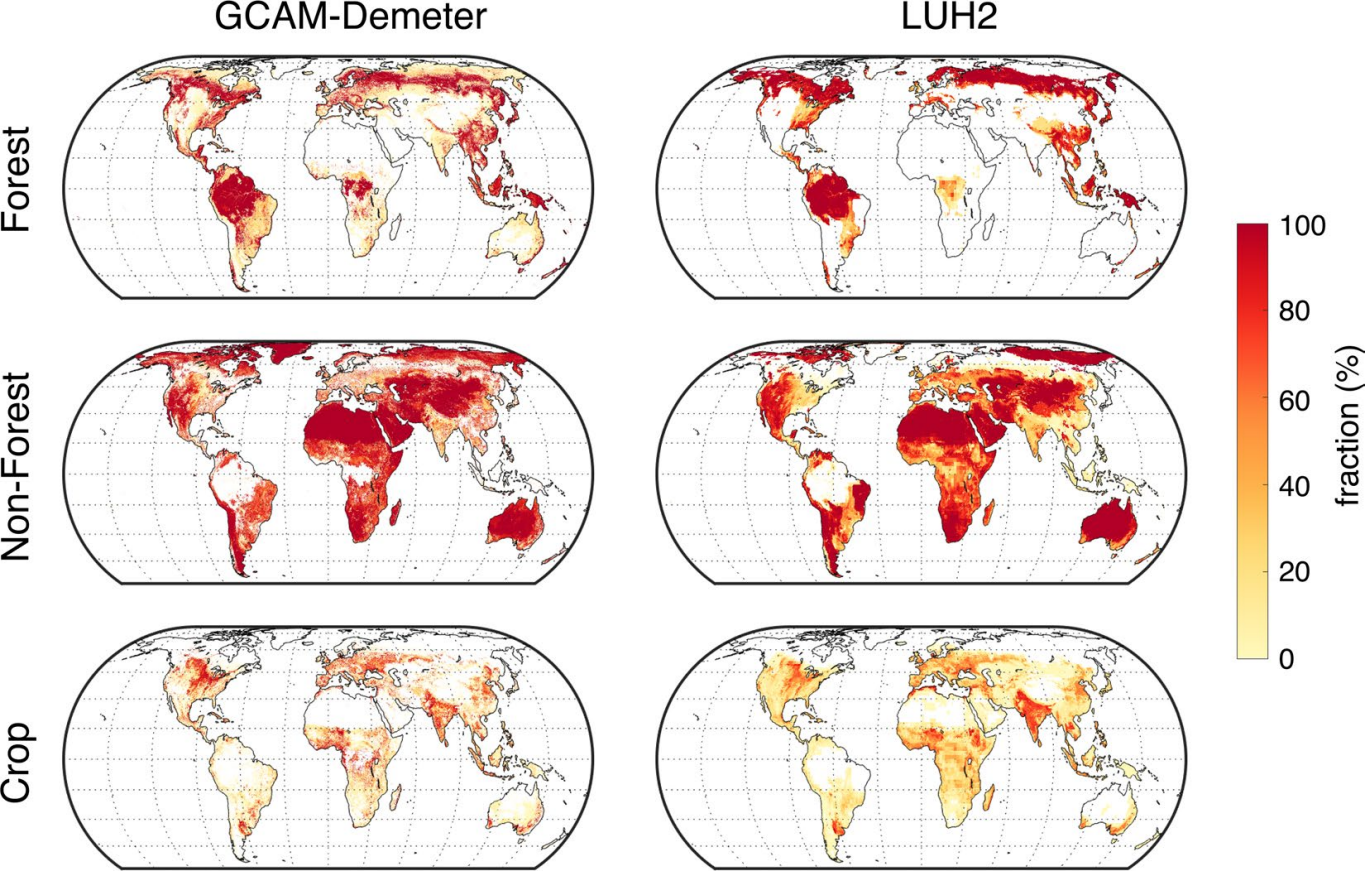
Comparison of land use spatial pattern of the three broad land cover types between our dataset and LUH2 in 2100 under SSP4 RCP6.0. Definitions of the three broad land cover types are the same as those in Fig. 3.

**Fig. 5:**
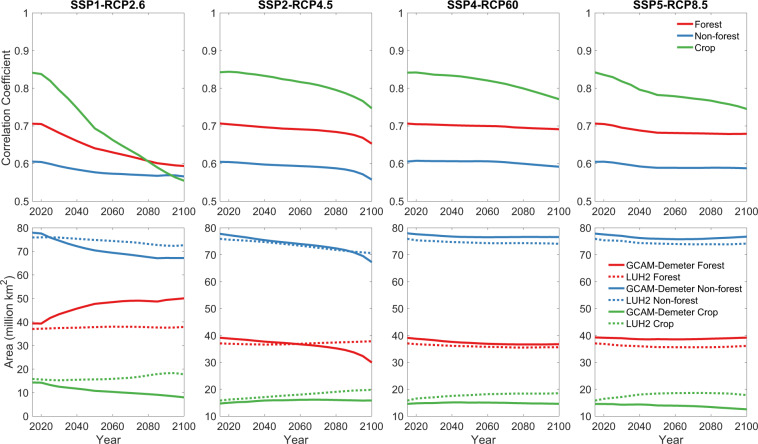
Correlation coefficients (top panels) and global area (bottom panels) between the LUH2 data and the presented dataset for three broad land cover types (Forest, Non-forest, and Crop) over 2015–2100 under SSP 1 RCP 2.6, SSP 2 RCP 4.5, SSP4 RCP 6.0, and SSP5 RCP 8.5 scenarios.

We further compared the two datasets under their overlapped SSP-RCP scenarios (SSP1 RCP2.6, SSP2 RCP4.5, SSP4 RCP6.0 and SSP5 RCP8.5). Taking the data in 2100 under SSP4 RCP6.0 as an example (Fig. 4), we find visually similar difference between the two datasets as that in 2015 (Fig. 3). More quantitative comparisons are shown in Fig. 5. The Pearson’s correlation coefficients between our dataset and LUH2 are the highest for ‘Crop’, then for ‘Forest’, and the lowest for ‘Non-forest’. The relatively weaker correlations for ‘Non-forest’ are due to the fact that our dataset does not distinguish snow ice. In general, the correlation coefficients show decreasing trend under all scenarios, particularly under SSP1 RCP2.6, indicating the discrepancy between the two datasets are getting larger over time. In addition, the global areas of the three broad land cover types from our dataset and LUH2 are at the similar level, but they are not fully consistent and the inconsistency changes over time. Such discrepancies are most likely due to that LUH2 use projections of a different model for each of the scenarios, e.g., IMAGE^11^ for SSP1 RCP2.6, MESSAGE-GLOBIOM^13^ for SSP2 RCP4.5, SSP4 RCP6.0 for GCAM, and REMIND-MAGPIE^12^ for SSP5 RCP8.5, while our dataset is solely based on GCAM. However, LUH2 used land cover projections from an older version of GCAM^42^, and we have adjusted the original GCAM projections to match the base map in our approach (see methods). Both factors may contribute to the discrepancy under SSP4 RCP6.0, although it is relatively smaller than the discrepancy under the other scenarios.

### Uncertainties due to the driving GCMs

**Fig. 6:**
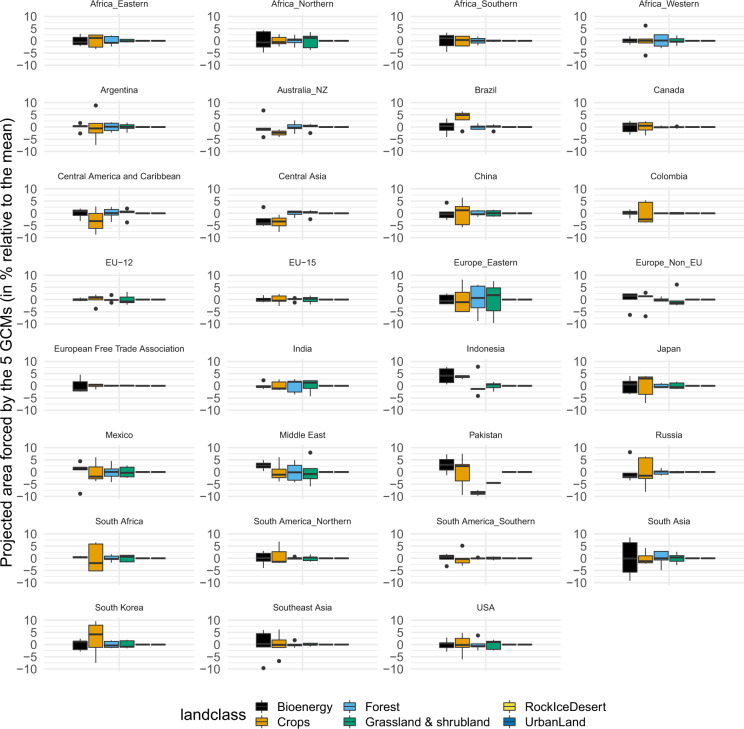
Uncertainty of GCAM-projected land cover areas in 2100 under SSP4 RCP6.0 due to the driving GCMs in each of the GCAM regions (indicated by the title of each panel). For illustration clarity, GCAM land types were grouped into broader types, such as ‘Bioenergy’ (GLT# 21–24, refer to Table 1), ‘Crops’ (GLT# 1–20, 25–31), ‘Forest’ (GLT# 33–35); ‘Grassland & shrubland’ (GLT# 36–38), ‘RockIceDesert’ (GLT# 39), and ‘Urbanland’ (GLT# 32).

The climate forcing provided by the five GCMs drives a few key components in GCAM, such as water supply, agricultural productivity, hydropower capacity changes, and building energy demands^31^. Thus, variations of climate forcing can introduce uncertainty in GCAM-projected (and thus the downscaled) land use under each of the SSP-RCP scenarios. Below we demonstrated the areal uncertainties in 2100, for each of the GCAM regions and broad land type groups due to the GCM forcing using the SSP4 RCP6.0 scenario as an example (Fig. 6). We found that the use of different GCMs can bring some uncertainties to the resulting area sharing of different land cover types in each of the regions (box plots show distributions of percentage deviations from the mean projected area forced by the five GCMs). ‘RockIceDesert’ and ‘Urbanland’ has no variation because no changes are projected by GCAM for these two land types. The uncertainty ranges differ with regions. For example, the 25^th^ and 75^th^ percentile of the areal variations of ‘Forest’ land types in ‘Africa_Eastern’ are −0.9% (0.9% less than the mean) and 1.8% (1.8% more than the mean), while they are −3.4% and 5.5% respectively in the region ‘Europe_Eastern’. Overall the uncertainty ranges (i.e., the maximum minus the minimum) are small, but for some certain land types and regions, the uncertainty ranges can be as large as 10%, particularly for ‘Crops’, such as those in ‘China’, ‘Europe_Eastern’, ‘Japan’, ‘Pakistan’, ‘Russia’, ‘South Africa’ and ‘South Korea’. Thus, while we think the mean land use driven by the five GCMs are sufficiently useful in most cases, we include both the spatially explicit mean and the standard deviation land use records at each time step in our dataset to represent the uncertainties due to GCM drivers.

## Usage Notes

The presented dataset includes global gridded land use and land cover change projections in the 21^st^ century under fifteen diverse SSP-RCP scenarios. The land cover types (in FLTs) were grouped following the classification system of a representative ESM, thus can be conveniently applied in simulations of a wide range of existing ESMs. Since the dataset is provided at a relatively high spatial resolution (0.05 degree), it offers good flexibility of ESM simulations at various spatial resolutions, which are typically at coarser resolutions. However, several important limitations have to be considered for correct use of this dataset.

First, since urban land is static in the current version of GCAM, the urban area in the downscaled products does not change over time. Additional consideration of urban dynamics will need to be taken into account for certain ESM simulations. Second, the land use and land cover change provided in this dataset is directly driven by human impacts, and there was no consideration of the biophysical response of vegetation (i.e., the dynamic vegetation) to climate change in the development of this product. Including the biophysical dynamic vegetation requires further development of GCAM in the future. Third, since the land use projections are generated by a single model, we cannot assess the uncertainty associated with the model structure. Future research should consider applying Demeter in conjunction with models other than GCAM.

## Data Availability

The source code of GCAM and Demeter used in this paper s available at 10.5281/zenodo.3713432^43^ and 10.5281/zenodo.3713378^44^, respectively.

## References

[1] Kalnay E, Cai M (2003). Impact of urbanization and land-use change on climate. Nature.

[2] Foley JA (2005). Global Consequences of Land Use. Science (80-.)..

[3] Lawrence DM (2016). The Land Use Model Intercomparison Project (LUMIP) contribution to CMIP6: rationale and experimental design. Geosci. Model Dev..

[4] Eyring V (2016). Overview of the Coupled Model Intercomparison Project Phase 6 (CMIP6) experimental design and organization. Geosci. Model Dev..

[5] Bonan GB (2008). Forests and Climate Change: Forcings, Feedbacks, and the Climate Benefits of Forests. Science (80-.)..

[6] Lawrence PJ, Lawrence DM, Hurtt GC (2018). Attributing the Carbon Cycle Impacts of CMIP5 Historical and Future Land Use and Land Cover Change in the Community Earth System Model (CESM1). J. Geophys. Res. Biogeosciences.

[7] Malyshev S, Shevliakova E, Stouffer RJ, Pacala SW (2015). Contrasting Local versus Regional Effects of Land-Use-Change-Induced Heterogeneity on Historical Climate: Analysis with the GFDL Earth System Model. J. Clim..

[8] Friedl MA (2002). Global land cover mapping from MODIS: algorithms and early results. Remote Sens. Environ..

[9] Hansen MC, Defries RS, Townshend JRG, Sohlberg R (2000). Global land cover classification at 1 km spatial resolution using a classification tree approach. Int. J. Remote Sens..

[10] Hurtt G (2011). Harmonization of land-use scenarios for the period 1500–2100: 600 years of global gridded annual land-use transitions, wood harvest, and resulting secondary lands. Clim. Change.

[11] Stehfest, E. *et al*. *Integrated Assessment of Global Environmental Change with IMAGE 3.0 - Model description and policy applications*. (PBL Netherlands Environmental Assessment Agency, 2014).

[12] Popp A (2014). Land-use protection for climate change mitigation. Nat. Clim. Chang..

[13] Havlík P (2011). Global land-use implications of first and second generation biofuel targets. Energy Policy.

[14] Fujimori S (2017). SSP3: AIM implementation of Shared Socioeconomic Pathways. Glob. Environ. Chang..

[15] Riahi K (2017). The Shared Socioeconomic Pathways and their energy, land use, and greenhouse gas emissions implications: An overview. Glob. Environ. Chang..

[16] Vuuren DP (2011). The representative concentration pathways: an overview. Clim. Change.

[17] O’Neill BC (2016). The Scenario Model Intercomparison Project (ScenarioMIP) for CMIP6. Geosci. Model Dev..

[18] O’Neill BC (2017). The roads ahead: Narratives for shared socioeconomic pathways describing world futures in the 21st century. Glob. Environ. Chang..

[19] Box EO (1996). Plant functional types and climate at the global scale. J. Veg. Sci..

[20] Woodward FI, Cramer W (1996). Plant functional types and climatic change: Introduction. J. Veg. Sci..

[21] DeFries RS (1995). Mapping the land surface for global atmosphere-biosphere models: Toward continuous distributions of vegetation’s functional properties. J. Geophys. Res. Atmos..

[22] Lawrence DM (2019). The Community Land Model Version 5: Description of New Features, Benchmarking, and Impact of Forcing Uncertainty. J. Adv. Model. Earth Syst..

[23] Edmonds, J. A. *et al*. In *Encyclopedia of Sustainability Science and Technology* (ed. Meyers, R. A.) 5398–5428 (Springer, 2012).

[24] Kim, S. H., Edmonds, J., Lurz, J., Smith, S. J. & Wise, M. The objECTS Framework for integrated Assessment: Hybrid Modeling of Transportation. *Energy J*. **Hybrid Modeling**, 63–92 (2006).

[25] Calvin K (2017). The SSP4: A world of deepening inequality. Glob. Environ. Chang..

[26] Calvin K (2019). GCAM v5.1: representing the linkages between energy, water, land, climate, and economic systems. Geosci. Model Dev..

[27] Wise M, Calvin K, Kyle P, Luckow P, Edmonds J (2014). Economic and physical modeling of land use in gcam 3.0 and an application to agricultural productivity, land, and terrestrial carbon. Clim. Chang. Econ..

[28] Kim SH (2016). Balancing global water availability and use at basin scale in an integrated assessment model. Clim. Change.

[29] Turner SWD, Hejazi M, Yonkofski C, Kim SH, Kyle P (2019). Influence of Groundwater Extraction Costs and Resource Depletion Limits on Simulated Global Nonrenewable Water Withdrawals Over the Twenty-First Century. Earth’s Futur..

[30] Graham NT (2018). Water Sector Assumptions for the Shared Socioeconomic Pathways in an Integrated Modeling Framework. Water Resour. Res..

[31] Graham NT (2020). Humans drive future water scarcity changes across all Shared Socioeconomic Pathways. Environ. Res. Lett..

[32] Chen M, Vernon CR, Huang M, Calvin KV, Kraucunas IP (2019). Calibration and analysis of the uncertainty in downscaling global land use and land cover projections from GCAM using Demeter (v1.0.0). Geosci. Model Dev..

[33] Vernon CR (2018). Demeter – A Land Use and Land Cover Change Disaggregation Model. J. Open Res. Softw..

[34] Page YLe, West TO, Link R, Patel P (2016). Downscaling land use and land cover from the Global Change Assessment Model for coupling with Earth system models. Geosci. Model Dev..

[35] Popp A (2017). Land-use futures in the shared socio-economic pathways. Glob. Environ. Chang..

[36] Warszawski L (2014). The Inter-Sectoral Impact Model Intercomparison Project (ISI–MIP): Project framework. Proc. Natl. Acad. Sci..

[37] West TO, Page YLe, Huang M, Wolf J, Thomson AM (2014). Downscaling global land cover projections from an integrated assessment model for use in regional analyses: results and evaluation for the US from 2005 to 2095. Environ. Res. Lett..

[38] Chen M, Vernon CR (2020). Zenodo.

[39] Di Vittorio AV (2014). From land use to land cover: restoring the afforestation signal in a coupled integrated assessment–earth system model and the implications for CMIP5 RCP simulations. Biogeosciences.

[40] Di Vittorio, A. V. *et al*. Quantifying the Effects of Historical Land Cover Conversion Uncertainty on Global Carbon and Climate Estimates. **45**, 974-982 *Geophys. Res. Lett*. (2018).

[41] Chen M (2020). Pacific Northwest National Laboratory 2; PNNL.

[42] Hurtt, G. C. *et al*. Harmonization of Global Land-Use Change and Management for the Period 850-2100 (LUH2) for CMIP6. *Geosci. Model Dev. Discuss*. 1–65 (2020).

[43] Calvin K (2020). Zenodo.

[44] Vernon C, Chen M (2020). Zenodo.

